# *Cannabis sativa*: Interdisciplinary Strategies and Avenues for Medical and Commercial Progression Outside of CBD and THC

**DOI:** 10.3390/biomedicines9030234

**Published:** 2021-02-26

**Authors:** Jackson M. J. Oultram, Joseph L. Pegler, Timothy A. Bowser, Luke J. Ney, Andrew L. Eamens, Christopher P. L. Grof

**Affiliations:** 1 Centre for Plant Science, University of Newcastle, University Drive, Callaghan, NSW 2308, Australia; Jackson.Oultram@uon.edu.au (J.M.J.O.); Joseph.Pegler@uon.edu.au (J.L.P.); Andy.Eamens@newcastle.edu.au (A.L.E.); 2 CannaPacific Pty Ltd., 109 Ocean Street, Dudley, NSW 2290, Australia; tim@cannapacific.com.au; 3 School of Psychological Sciences, University of Tasmania, Hobart, TAS 7005, Australia; luke.ney@utas.edu.au

**Keywords:** *Cannabis sativa* (*Cannabis*), cannabinoids, tetrahydrocannabinol (THC), cannabidiol (CBD), cannabinoid receptors (CB_1_ and CB_2_), *Papaver somniferum* (opium poppy), secondary metabolites

## Abstract

*Cannabis sativa* (*Cannabis*) is one of the world’s most well-known, yet maligned plant species. However, significant recent research is starting to unveil the potential of *Cannabis* to produce secondary compounds that may offer a suite of medical benefits, elevating this unique plant species from its illicit narcotic status into a genuine biopharmaceutical. This review summarises the lengthy history of *Cannabis* and details the molecular pathways that underpin the production of key secondary metabolites that may confer medical efficacy. We also provide an up-to-date summary of the molecular targets and potential of the relatively unknown minor compounds offered by the *Cannabis* plant. Furthermore, we detail the recent advances in plant science, as well as synthetic biology, and the pharmacology surrounding *Cannabis.* Given the relative infancy of *Cannabis* research, we go on to highlight the parallels to previous research conducted in another medically relevant and versatile plant, *Papaver somniferum* (opium poppy), as an indicator of the possible future direction of *Cannabis* plant biology. Overall, this review highlights the future directions of cannabis research outside of the medical biology aspects of its well-characterised constituents and explores additional avenues for the potential improvement of the medical potential of the *Cannabis* plant.

## 1. Introduction

*Cannabis sativa* (*Cannabis*) is arguably one of the world’s most versatile crops. While the genetic origin and evolution of *Cannabis* is a long-standing and heavily debated topic [1,2,3,4], in broad terms, today, *Cannabis* can be separated into two distinct categories, specifically ‘hemp’ and ‘marijuana’. Much like other agricultural crop commodities, *Cannabis* has been domesticated and bred for thousands of years to produce phenotypic and/or chemotypic traits of value to humans [2,3,4,5]. The chemotypic distinction between hemp and marijuana predominantly stems from the abundance of the principal psychoactive cannabinoid, Δ^9^-tetrahydrocannabinol (THC), present in the plant as the acidic form, Δ^9^-tetrahydrocannabinolic acid (THCA) [6]. To be considered hemp, *Cannabis* must possess a low percentage of THC relative to the total dry weight of flowers, with this low THC percentage varying from country to country. In order to be legally cultivated as hemp, the cultivated plants must possess less than 0.3% THC (*w/w*) in Canada [4,7] and China [8], whereas since 2001, the European Union determined that the THC content (*w/w*) of hemp must be below 0.2% [6].

Hemp has traditionally been bred as a source for textile products due to the strong, elongated bast fibres present in the phloem of the stem. More recently, the elevated cellulosic content of hemp cell walls has garnered interest in the plant as a source for the development of sustainable biofuel production [6]. Hempseed, and hempseed oil, have historically been utilised as a food source, with more contemporary research revealing their unique dietary value. In particular, the essential polyunsaturated fatty acids (PUFAs), linoleic acid (LA) and linolenic acid (LNA), comprise 50–70% and 15–25% of the total fatty acid content of hempseed, respectively; a 3:1 ratio promoted as nutritionally optimal [9,10,11,12,13]. PUFAs found in hempseed oil are incorporated into phospholipid bilayers and are integral to membrane fluidity and the maintenance of its permeability [14]. Moreover, the two proteins, edestin and albumin found in hempseed, contain rich amino acid profiles comparable to that of high-quality soybean and egg white [15]. Given the functions and importance of both fatty and amino acids, hempseed and hempseed oil may have some potential, albeit minor, for reducing the incidence of certain diseases, while in parallel conferring a range of health benefits [15,16,17]. Alternatively, marijuana has traditionally been bred for its recreational intoxication properties derived from the THCA-containing resin produced on the protruding secretory hair-like structures known as trichomes which are predominantly located on female reproductive parts of the *Cannabis* plant [18,19]. The sticky resin produced from these specialised epidermal glands is a rich mix of cannabinoid and non-cannabinoid constituents, numbering at least 104 and 441, respectively [20,21]. Most recently, two novel cannabinoids, namely Δ^9^-tetrahydrocannabiphorol (Δ^9^-THCP) and cannabidiphorol (CBDP), near identical in structure to THC and cannabidiol (CBD), respectively, were identified [22]. Notably, Δ^9^-THCP was demonstrated to possess higher cannabimimetic activity than THC, and its recent discovery is therefore postulated as a potential candidate cannabinoid responsible for variation in pharmacological properties observed in uncharacterised *Cannabis* varieties. This also identifies the likelihood of secondary metabolites present in *Cannabis* resin that remain to be discovered.

In addition to possessing a range of phenotypic and chemotypic traits of interest to the textile, medicinal, food and energy industries as an agricultural crop, *Cannabis* is extremely versatile and hardy, hence the application of the colloquial term for this species, ‘weed’. The phenotypic flexibility of *Cannabis* provides it with the capacity to adapt and survive a range of abiotic and biotic insults, such as drought [23], heavy metal stress [24], high temperature [25], poor soil nutrient content [3], high plant density [26], and stem damage from the larva of *Ostrinia nubilalis*, the European corn borer [27]. Tolerance to a range of abiotic stress conditions is exemplified by the tap root of *Cannabis* which is able to adapt to highly variable edaphic conditions, either penetrating deep (greater than 2 metres) into dry soil, or developing an extensive lateral root network in response to its growth in soil that has a high moisture content [26]. Further, the widespread legalisation of medicinal application and recreational use of *Cannabis* is driving the growth of diverse research programs encompassing the broad scope, from plant breeding to clinical trials. In the United States of America (USA), for example, to date, 33 states have approved the medicinal use of *Cannabis*, while 14 states and territories have legalised the recreational use of marijuana by adults. At the federal level in the USA, however, *Cannabis* remains a ‘*Schedule I Substance*’. In direct contrast to the heavy legislation of *Cannabis* in the USA, its direct neighbour, Canada, legalised the use of *Cannabis* across the country in 2018 under the ‘*Cannabis Act*’ [28]. As the legislative approval of *Cannabis* use increases worldwide, there will be an increasing need for interdisciplinary research to characterise secondary metabolites of interest and to increase the production of *Cannabis* to meet the demand for medicinal and recreational products.

Currently, there exists an extant literature on the medical potential for the best characterised cannabinoids, THC and CBD [29,30,31,32,33,34]. Significantly less attention in medical research has been paid to the potential for the minor phytocannabinoids to treat illnesses, and there is still the need for methods to produce these cannabinoids cost-effectively for commercial production. In particular, the medical *Cannabis* industry faces significant challenges in multiple aspects of product development. For instance, THC is associated with multiple side effects, and furthermore, pharmaceutical-standard THC and CBD are expensive to produce. Due to these hurdles, many companies around the world which have attempted to capitalise on the increasing legality of *Cannabis* have been unsuccessful [35]. Therefore, here we review the current literature describing emerging research concerning the medical potential of the minor cannabinoids, as well as to outline the agricultural and production considerations that will be necessary to meet the needs of the growing medical market. Readers interested primarily in the effects of CBD and THC should consult any of the substantial reviews on these topics that are published elsewhere and referred to here in Section 2.2. It should also be noted that there are some recent review articles on the molecular targets of the minor cannabinoids [36,37], but to the best of our knowledge, no published review of the current literature has combined this research with the potential for improving *Cannabis* yield and extraction efficacy to make these possibilities economically and logistically pragmatic. This review therefore presents a novel, interdisciplinary perspective on the practical possibilities for improving the *Cannabis* species for its utilisation in the cannabinoid industry in the near future.

## 2. The Endocannabinoid System and Its Associated Molecular Targets

### 2.1. An Overview of the Endocannabinoid System

The discovery of the endogenous cannabinoid system followed the initial isolation [38] and synthesis [39] of the primary psychoactive compound in *Cannabis*, THC. Following on from this in the late 1980s, and into the early 1990s, two cannabinoid receptors, CB_1_ and CB_2_, were identified [40,41]. Surprisingly, it was discovered that CB_1_ was highly abundant in the central nervous system (CNS), and in the CNS, CB_1_ is one of the most profuse G protein-coupled receptors [42]. The identification of these two CB receptors subsequently led to the discovery of an endogenous receptor ligand termed arachidonylethanolamide (anandamide), a receptor ligand accurately predicted to exist based on the presence of the CB receptors themselves [43]. A second receptor ligand, 2-arachidonoylglycerol (2-AG) was later identified [44,45]. Anandamide and 2-AG are both synthesised from arachidonic acid. Synthesis of anandamide is complex, and therefore remains to be elucidated, though it is thought to occur largely via the cleavage of arachidonic acid by a phospholipase D from its membrane precursor, N-arachidonoyl phosphatidylethanolamine [46]. The synthesis of 2-AG occurs following the conversion of diacylglycerol by the metabolic enzyme, diacylglycerol lipase (DAGL). Hydrolysis of anandamide occurs via the enzyme activity of fatty acid amide hydrolase (FAAH), whereas 2-AG is hydrolysed by both FAAH and monoacylglycerol lipase (MAGL) [47]. Inhibition of these enzymes increases anandamide and 2-AG concentrations and has therapeutic potential [48,49,50]. Similarly, it is possible that modulation of precursory compounds of anandamide and 2-AG may have therapeutic potential [51].

Previous investigations into CB receptor distribution within the fetal, neonatal and adult human brain revealed that the CB receptors were primarily localised to areas responsible for; (1) higher cognitive function; (2) movement, and; (3) control of sensory and motor functions of the autonomic nervous system [52]. Protein crystallisation has revealed the structure of CB_1_ [53] and CB_2_ [54] to assist in the characterisation of the molecular binding of ligands, such as THC, and potentially other key cannabinoids, both naturally or synthetically produced. Using radiolabelled synthetic cannabinoids, it was shown that the highest density of cannabinoid binding, and thus CB receptor localisation, appeared in the basal ganglia, hippocampus and cerebellum [42]. Cannabinoids were shown to function on hippocampal presynaptic receptors, via regulating the release of γ-aminobutyric acid (GABA) to modulate higher cognitive functions, while also increasing the activity of p38 mitogen-activated protein kinases [55,56]. Similarly, GABA modulation in the basal ganglia, specifically the presynaptic striatal projection neuron axons and their termini, was found to be stimulated to differing degrees by either endocannabinoids or synthetic cannabinoids [57,58]. The binding of the CB_1_ receptor by both endogenous and exogenous cannabinoids also modulates excitatory synaptic transmission in Purkinje cells located in the cerebellum [59,60,61,62]. Crucially, endocannabinoid signalling was recognised as the mediatory secondary messenger responsible for long-term potentiation, and depression [49,63], which are both fundamental to the control of synaptic transmission. CB_1_ receptors and endocannabinoid signalling also interacts with other systems in the brain, such as the dopaminergic [64], and glucocorticoid [65] pathways, to modulate stress response and associative learning processes.

While early understanding of receptor distribution suggested exclusive ‘central’ aggregation in specific regions of the brain, it is now understood that there is a more extensive presence of CB_1_ type receptors in peripheral tissues. Two CB_1_ receptor isoforms have since been identified, both of which display distinct expression patterns in pancreatic β-cells and liver hepatocytes [66]. Antagonism of peripheral CB receptors located in skeletal muscles was shown to trigger glucose uptake, while simultaneously initiating lipid mobilisation in white adipose tissue [67]. Though the protein expression pattern of CB_1_ does show some overlap with CB_2_ in peripheral tissues, and conversely some CB_2_ receptors are cerebrally positioned [68,69,70,71,72], peripheral receptors are predominantly CB_2_ type receptors. Analysis of *CB_2_* transcript levels has previously revealed its expression in the tonsils, spleen, and peripheral blood mononuclear cells, where further cell isolation showed detectable *CB_2_* transcript levels in polymorphonuclear neutrophils (PMN), T4 cells, T8 cells, natural killer (NK) cells, macrophages, and B cells. However, at the protein level, the CB_2_ receptor appears to be restricted to B cells [73]. Similarly, CB_2_ receptor binding has been observed in other immune system regions, namely the lymph node cortex, as well as in the Peyer’s patches, which are areas of B lymphocyte aggregation [74]. The expression and/or localisation of functional CB_2_ protein has also been reported for mast cells, modulating their initial activation, or downregulating their activity post their initial activation, an activity change which can in turn provoke an anti-inflammatory response [75]. Anandamide and 2-AG, as well as their metabolic enzymes, are detectable in blood [76,77], hair [78,79,80], saliva [81,82,83], breast milk [84,85], and reproductive fluids [84,86]. Compounded with the peripheral anti-inflammatory response, CB_2_ receptor agonists can mediate peripheral antinociception without the psychotropic CNS effects associated with phytocannabinoid CB_1_ receptor binding [87,88]. This characteristic of exerting medically beneficial effects, while simultaneously avoiding any psychotropic responses, is likely to form a key focus of future cannabinoid research.

### 2.2. The Expanded Cannabinoid System and Its Less Characterised Receptors

It has been clearly demonstrated that the collective effects of cannabinoid administration cannot be explained solely by the presence of CB receptors. Conversely, it has been increasingly recognised that cannabinoids have the potential to affect other molecular targets and receptor types, particularly given their role as presynaptic secondary messengers on various neuron species [89,90] (Table 1). One such receptor is the G protein-coupled receptor (GPCR), GPR55, with the *GPR55* transcript identified in the adrenals, jejunum, and ileum in mammalian systems [91]. Studies on canine, rat and mouse gastrointestinal systems collectively suggest that GPR55 may be involved in smooth muscle contractions and colonic motility, especially when activated by CBD, pointing to a potential target for treatment of some gastrointestinal disorders [92,93,94,95]. Human embryonic kidney 293 (HEK293) cells expressing the GPR55 protein have been assessed for their response when treated with the lysolipid, L-α-lysophosphatidylinositol (LPI), as well as following their treatment with endogenous, synthetic or phytocannabinoids. LPI was found to induce phosphorylation of the protein, extracellular signal-related kinase (ERK) in GPR55-expressing cells, while also initiating a transient Ca^2+^ signal involved in downstream messaging and intracellular processing [96]. The degree of elevation in the concentration of Ca^2+^ increases in HEK293 cells when mediated by GPR55-phospholipase C coupling varied depending on whether THC, anandamide, methanandamide or the CB_2_ agonist, JWH015 was administered [97]. However, there was no Ca^2+^ response initiated by CBD, the CBD regioisomer abnormal CBD, the endogenous cannabinoids, 2-arachidonoylglycerol and *O*-arachidonoyl ethanolamine, or the synthetic cannabinoids, WIN55,212-2 and CP55,940 [97]. Beyond Ca^2+^ transients, cannabinoid ligand interaction with the GPR55 receptor promotes ERK phosphorylation, as well as the varied activation of cyclic adenosine monophosphate (cAMP) response element binding protein (CREB), nuclear factor-_κ_B (NF-_κ_B) and nuclear factor of activated T-cell (NFAT) transcription factors, the latter two of which are involved in inflammation of endothelial cells and irritable bowel syndrome (IBS) [98,99,100,101]. The *GPR55* transcript can also be found in the basal ganglia, hippocampus, forebrain, cerebellum, cortex and large dorsal root ganglion (DRG) [97,102,103,104]. The expression of *GPR55* in these tissues significantly broadens the potential for its therapeutic application. For instance, activation of the GPR55 receptor by THC enhances neuronal excitability and reduces the M-type potassium current, which when combined with the expression pattern of *GPR55* in the large DRG, indicates a nociceptive role [97]. Inflammatory pain was modulated by abnormal CBD through GPR55 antagonism in acute arthritis models in rats [105]. Evidence of pro-nociception was observed in rats when the abundance of GPR55-dependent Ca^2+^ increased in periaqueductal grey neurons and which preceded a pain threshold reduction [106]. However, another study [107] reported that GPR55 knockout mice show no difference to wild-type mice in neuropathic pain models.

Another seven-transmembrane G protein-coupled receptor, termed GPR18, was first identified in canine gastric mucosa and a human colonic cancer cell line, with a high abundance of the *GPR18* transcript detected in human testis and spleen tissue [108]. The candidate ligand was later suggested to be *N*-arachidonoyl glycine (NAGly), an anandamide metabolite, which was first detected when GPR18-expressing cell lines, including the L929, K562 and Chinese hamster ovary (CHO) cell lines produced, high levels of intracellular Ca^2+^ and inhibited the production of cAMP following NAGly exposure [109]. In addition, quantitative real-time PCR analysis revealed high levels of *GPR18* expression in peripheral lymphocytes, further supporting the suggestion of a role in immune system function [109].

The transient receptor potential vanilloid (TRPV) channels are a subfamily of transmembrane ligand-gated ion channels that mediate signal transduction processes initiated by a broad range of noxious stimuli in animals, with the TRPVs, TRPV1 through to TRPV4, activated to varying degrees via cannabinoid application. TRPV expression in several human tissues and the documented role of TRPVs in human disease is a current avenue of interest. The capsaicin and temperature (~42 °C) responsive TRPV1, displays an ambiguous expression profile. However, the weight of evidence suggests that its expression domain is rather broad in animal systems. Specifically, the TRPV1 protein was observed to be localised to the dorsal root and trigeminal ganglions [110], thermoregulatory tissue smooth muscle cells [111], urothelial cells [112], corneal fibroblasts [113], and a broad distribution profile in the brain, including the hippocampus, cortex and olfactory bulb [114]. Sharing 50% sequence identity to TRPV1, TRPV2 has been demonstrated to respond to high-intensity thermal stimuli (~52 °C). However, unlike TRPV1, TRPV2 is insensitive to capsaicin [115]. Given its sensory involvement, TRPV2 localisation in the ganglia is unsurprising. However, TRPV2 is also localised to the brain, lung, spleen, intestine, mast cells and lymphocytes [115,116,117,118], which, when considered together, infers additional TRPV2 function beyond heat sensing, and by extension, activation by non-thermal receptor modulators. The initiation of signal cascades via TRPV2 are potentially involved in diseases and physiological responses including cancer [119], the innate and adaptive immune responses [116,117,120,121], cardiomyopathy [122,123], muscular dystrophy [124,125], and insulin secretion response [126,127,128].

The cannabinoid-responsive TRPVs, TRPV3 and TRPV4, are also temperature sensitive proteins. The responsive temperature range (27–40 °C) for these two receptors is below that of TRPV1 and TRPV2, but they do closely overlap with one another [129,130,131,132]. Their thermosensory involvement localises these two TRPVs to keratinocytes, where they sense warmth on the skin and transmit a signal to nearby neurons [133,134,135,136,137,138]. In the tongue and nasal epithelium, TRPV3 is activated by the ‘pungent’ carvacrol as well as by thymol and camphor [133,139], whereas the mevalonate (MVA) pathway product and cannabinoid/terpenoid precursor, isopentenyl diphosphate (IPP), has been shown to inhibit TRPV3 activity [140]. TRPV4, in association with aquaporin 5 (AQP5), is additionally involved in osmosensing and regulatory volume decrease in cells following swelling in hypotonic environments [141,142,143,144]. Located in the brain [145,146], kidneys [147], CNS [148], and endocardium [149], TRPV4 activity is also modulated by phorbol esters and arachidonic acid expanding its activation beyond physical stimuli [150,151].

In addition to the vanilloid subtype of the transient receptor potential channels are the melastatin and ankyrin subtypes. Of the melastatin type, transient receptor potential melastatin 8 (TRPM8) is a cold/menthol-responsive channel located in the DRG and trigeminal ganglia [152,153]. Of the ankyrin subtype, transient receptor potential ankyrin 1 (TRPA1) acts similarly to TRPM8 in response to cold stimuli covering a similar temperature range (~8–28 °C). However, it is suggested that TRPA1 contributes to sensation of lower temperatures, and is also similarly localised in sensory neurons [154,155,156,157]. TRPA1 is additionally activated by formalin and allyl isothiocyanates such as mustard oil [158,159], and has further been implicated in eliciting inflammatory pain [160,161,162,163].

Multiple other targets show notable interactions with the endocannabinoid system; however, a comprehensive description of all interactions is beyond the scope of this review. Briefly, other notable molecular interactions include glycine receptors with anandamide, and in addition, CBD and THC have also been shown to activate glycine receptors [164,165]. Further, THC appears to exhibit dose-dependent effects on glycine receptor activation [166]. The activation of peroxisome proliferator-activated receptors (PPAR), in particular the α and γ subtypes, is responsible for many of the metabolic, analgesic, neuroprotective, and other health-related benefits of cannabinoids [167]. Cannabinoids have also been shown to interact with serotonergic sites, particularly with the 5-HT_1A_ [168] and 5-HT_2A_ [169,170] receptors, and these interactions are strongly associated with disorders such as anxiety and post-traumatic stress [171,172]. Consequently, the spectrum of potential therapeutic applications is very broad for cannabinoids and would require a specifically dedicated and lengthy review in its own right. Currently lacking are robust, double-blind in vivo and clinical studies of the constituents of the broader cannabinoid profile that target specific diseases, and/or can be used to treat the symptoms of these diseases, possibly via targeting the interactions between cannabinoids and these other putative or lesser-known receptors.

**Table 1:** Receptor modulation by cannabinoids and studies outlining their potential involvement in disease treatment.

Receptor	Cannabinoid	Disease/Interaction	Study Type	Reference
**CB_1_**	Anandamide	Appetite	Murine models	[173,174]
	Met-F-AEA	Thyroid cancer	in vitro human	[175]
	THCB _(PA)_	Pain	Murine models	[176]
	THC _(PA)_	Epilepsy	Murine models	[177]
		Sleep	Various studies	[178]
	THCP _(Ag)_	Pain, anxiety, hypothermia, catalepsy	Murine models	[22]
	THCV _(^)_	Pain, anxiety, hypothermia, catalepsy	Murine models	[179,180]
		Parkinson’s disease	Murine models	[181]
		Obesity	Murine models	[182]
		Epilepsy	in vitro murine	[183]
	THC, WIN55,212-2, CP55, 940	Emesis	Animal models	[184,185,186,187,188]
	WIN55,212-2	Parkinson’s disease	Murine model	[189]
		Prostate cancer	in vitro human	[190]
	WIN55,212-2, JWH-133	Breast, lung cancer	in vitro human	[191,192]
**CB_2_**	CBC _(Ag)_	Inflammation	in vitro models	[193]
	CBG _(PA)_	Inflammatory bowel disease	Murine models	[194]
	HU-308, AM630	Parkinson’s disease	Murine models	[195,196]
	THCP _(Ag)_	Pain, anxiety, hypothermia, catalepsy	Murine models	[22]
	THCV _(^)_	Inflammation	Murine models	[180]
**CB_2_**	THCV _(^)_	Parkinson’s disease	Murine models	[181]
		Pain, anxiety, hypothermia, catalepsy	Murine models	[179]
	WIN55,212-2	Prostate cancer	in vitro human	[190]
	WIN55,212-2, JWH-133	Breast, lung cancer	in vitro human	[191,192]
**GPR55**	Abnormal CBD	Parkinson’s disease	Murine models	[103]
**GPR55**	Abnormal CBD	Pain/arthritis	Murine models	[105]
	CBD _(An)_	Gastrointestinal disorders	Canine, murine models	[93,94,95,96]
	CBDV _(An)_	Rett syndrome	Murine models	[197]
		LPI inhibitor	in vitro	[198]
	THC, anandamide, JWH015	Pain	in vitro HEK239	[97]
**TRPV1**	CBDV _(Ag)_	Anti-seizure	in vitro HEK239	[199]
	CBG _(Ag)_, CBGV, CBD _(Ag)_, CBDV _(Ag)_, THCV _(Ag)_	Receptor desensitisation	in vitro HEK239	[200]
**TRPV2**	CBD _(Ag)_, CBGV, CBG _(Ag)_, THCV _(Ag)_, CBDV _(Ag)_, CBN _(Ag)_	Receptor desensitisation	in vitro HEK239	[200]
**TRPV3**	CBGV, CBGA _(Ag)_	Receptor desensitisation	in vitro HEK239	[201]
**TRPV4**	CBGV, CBGA, CBN, CBG	Receptor desensitisation	in vitro HEK239	[201]
**TRPM8**	CBG _(An)_, CBC _(An)_, CBD _(An)_, CBDV _(An)_, THC _(An)_, THCA _(An)_	Colorectal cancer	in vitro model	[200,202,203]
**TRPA1**	CBC _(Ag)_, CBN _(Ag)_, THC _(Ag)_, THCV _(Ag)_, THCA _(Ag)_, CBDA, CBG _(Ag)_	Receptor desensitisation	in vitro HEK239	[200,202]
	CBDV _(Ag)_	Ulcerative colitis	in vitro human	[204]
		Muscular dystrophy	in vitro studies	[205]

PA = Partial Agonist, Ag = Agonist, ^ = Dose Dependent, An = Antagonist.

### 2.3. Examples of the Potential Medicinal Use of Cannabinoids

While research into the cannabinoids and their role in human disease is still in its infancy, the field abounds in promising preliminary studies. Cannabinoids, both of the endo- and phytocannabinoid categories, have been demonstrated to provide protection against further neurodegeneration in lesioned neurons post-treatment with toxic doses of 6-hydroxydopamine, as well as the neuron degeneration linked to Parkinson’s disease [189,206]. Moreover, symptoms of dyskinesia associated with Parkinson’s disease and other movement disorders, originating from deficiencies in the cannabinoid receptor-rich basal ganglia in marmosets, and reserpine-treated rats, have been reduced by CB_1_ receptor stimulation-mediated suppression of involuntary motor behaviour [189,207,208,209,210]. Central nervous system activation of the CB_2_ receptor has exhibited promising results in combating the inflammation and oxidative stress of Parkinson’s disease which is associated with dopaminergic neuron loss in the substantia nigra pars compacta in nonhuman models [195,196].

Studies into the treatment of a variety of cancers through cannabinoid use have also proved valuable. For example, CB_1_ and CB_2_ activation by either endogenous or synthetic receptor ligands has inhibited prostate [190] and pancreatic [211] adenocarcinoma growth, as well as breast [191] and thyroid [175] tumour growth. Modulation of non-CB receptors by the minor cannabinoids is also under investigation for their role in the initiation of oncogenic signalling cascades that may induce the arrest of the cell cycle, or inhibit the growth of tumours [212]. Endocannabinoid-mediated breast cancer cell proliferation has been inhibited by a reduction in prolactin action at the receptor level [213], and CB_1_ and CB_2_ receptor activation has induced apoptosis of cancerous cells in the breast [191] and colon [214]. In non-small-cell lung cancer cell lines, treatment with agonists targeting CB_1_ and CB_2_, or specifically CB_2_, were demonstrated to induce apoptosis, and to attenuate chemotaxis, metastatic growth and development, metastatic proliferation, and angiogenesis [192]. Similarly, cannabinoid activity against vascularization was also observed in human grade glioma cells in mice, with CB_2_ activation reducing tumour angiogenesis by inhibiting vascular endothelial cell migration and the suppression of pro-angiogenic factors in tumour cells [215].

First alluded to over 40 years ago, the use of *Cannabis* as a treatment for epilepsy has garnered traction in recent years and several comprehensive reviews have recently described the efficacy of cannabinoids in the treatment and/or management of epilepsy [216,217,218]. Further evidence of the involvement of the endocannabinoid systems in seizure mitigation is suggested with inactivation of the endocannabinoid degrading, FAAH, with FAAH shown to reduce both kainic acid associated seizure activity, and synaptic decline and damage to cytoskeletal elements in the hippocampus of rat models [219,220]. A double-blind, placebo-controlled study of 218 patients in which CBD was administered at a dose of 10 and 20 mg per kg reduced the frequency of drop seizures in both children and adults with Lennox-Gastaut syndrome, when compared to conventional epilepsy treatment [221]. A similar double-blind, placebo-controlled study of 120 children with the epilepsy disorder, Dravet syndrome, saw a significant reduction in the frequency of convulsive seizures when treated with CBD, as compared with those administered the placebo [222]. In a retrospective, open-labelled study, Press et al. [223] reported improvements in seizure control and frequency reduction in paediatric patients using oral *Cannabis* extracts, as well as additional improvements in some off-target metrics, including alertness and motor skill usage also observed. Use of a THC extract has attenuated seizure duration and termination via the activation of CB_1_. However, inhibition of CB_1_ receptor activity has also been demonstrated to increase the frequency and duration of seizures in non-human models, findings which firmly identify a role for CB_1_ in seizure responses [177]. Indeed, transgenic CB_1_ overexpressing mice were reported to have reduced kainic acid-induced seizure severity and mortality with reduced hippocampal neuron damage [224]. While these examples suggest promise in the efficacy of cannabinoids, or the modulation of cannabinoid receptor activity against epilepsy, there currently remains deficiencies in access to data emerging from large, controlled clinical studies.

The treatment of Parkinson’s disease, cancer and epilepsy are persistently pursued and remain ‘high-value’ targets for researchers. However, the importance of treating other less deleterious ailments, or the treatment of the negative side effects that originate from the aggressive treatment strategies of major diseases such as cancer, chemotherapy for example, is not without utility. A suite of clinical trials have supported the ability of *Cannabis*-derived metabolite constituents to (1) act as effective antiemetics [184,185,186,187,188], (2) ease the spasticity symptoms associated with Motor Neuron Disease and Multiple Sclerosis [225], (3) stimulate appetite [173,174,226,227,228,229], (4) help regulate sleep patterns [178,230,231,232], (5) initiate analgesia [233,234,235,236], (6) act as an anxiolytic to alleviate the psychotic symptoms of schizophrenia [237,238,239,240,241], (7) treat anxiety and post-traumatic stress disorders [31,171,242], (8) be utilised as palliative care agents [243,244], (9) aid in the acute inflammatory response and its protracted recovery [245], and (10) mitigate the effects of opioid addiction [246,247].

A full review of the current understanding of cannabis in the medical sphere is beyond the scope of this review and has been published elsewhere [90,248]. Despite much of the current research remaining in the preliminary stages, requiring a greater amount of more stringent, double-blind studies, the medicinal promise of *Cannabis* is readily evident. Meta-analyses relating to the legitimacy of medical *Cannabis*, specifically the use of CBD and THC in control randomised trials, have been conducted. Studies surrounding the use of CBD indicate that the drug is well tolerated with minimal serious adverse side effects and drug–drug interactions [249]. CBD is described as effective in the treatment of refractory seizures, but scientifically stringent data are lacking to claim effectiveness for other indications, with concerns remaining about the quality control in drug preparation and long-term safety [250]. It has been noted that inconsistencies across current studies relating to dosage and administration methods limit the conclusions that can be drawn to direct medical intervention using CBD [251]. Currently, cannabinoid therapies for sleep quality and mental health-related disorders also suggest that while preliminary evidence may indicate positive outcomes, the collation of eligible studies provides insufficient evidence to suggest efficacy or promote usage until additional, and more stringent studies have been conducted [252,253]. Although more stringent studies on the effectiveness of cannabinoids to control pain and spasticity exist, additional comprehensive studies demonstrating improvements in the treatment of chemotherapy associated nausea, sleep disorders, weight gain, and Tourette’s syndrome, and which also note the risk of short-term adverse events of cannabinoid treatment, are still required [32].

## 3. The Cannabinoid and Terpene Pathways of *Cannabis*

**Figure 1:**
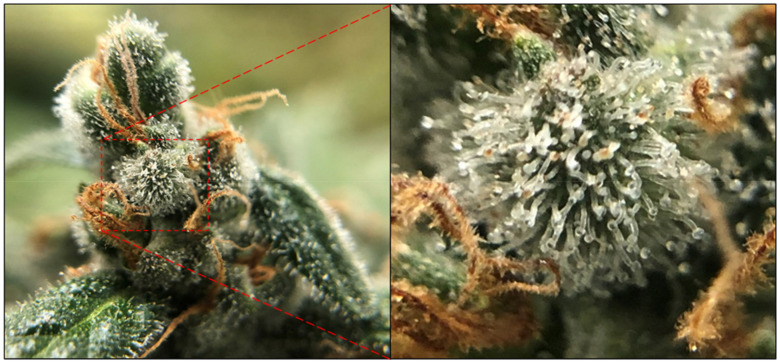
A close up of the female floral architecture of mature *Cannabis sativa* plants. The cannabinoid-containing glandular trichomes are visible in the magnified image, and are characterised by a globular head which is connected to the plant via a stalk. Colouration of the heads ranges from translucent, to a creamy white, to brown.

It is clear that modulation of the endocannabinoid system can be achieved outside of THC, CBD, and their CB receptors. Despite this, the majority of research conducted to date has sought to understand how these two cannabinoids interact with the various constituents of the expanded endocannabinoid system. However, significant knowledge exists concerning what further compounds can be extracted from *Cannabis* as well as an emerging understanding of how such compounds can be efficiently extracted from the *Cannabis* plant. To date, the most studied phytochemicals in *Cannabis* are the cannabinoids and terpenes. Together, these two classes of phytochemical comprise approximately 41% of the total number of known secondary metabolites identified in *Cannabis* [21,22]. Cannabinoid and terpenoid biosynthesis occurs in hair-like capitate stalked glandular trichomes [254,255], which cover the female floral organs, and exhibit a particularly high density on the bracts (a specialised leaf of the floral organs; Figure 1).

In trichome development, a protodermal cell is enlarged vertically out from the epidermis and subsequently undergoes anticlinal division, prior to a series of periclinal division events to create a secretory and auxiliary tier of cells atop the epidermal basal cells [256,257,258,259]. Additional division events develop the secretory tier of disc cells that form a cavity on the external surface of the trichome from a portion of the outer wall. This cavity then enlarges as the secretory vesicles that harbour a diverse payload of secondary metabolites are extruded into the expanding waxy cavity. Post their cellular release, the secreted vesicles disintegrate upon contact with the thickened outer cuticle wall to release their contents [256,257,258,259].

**Figure 2:**
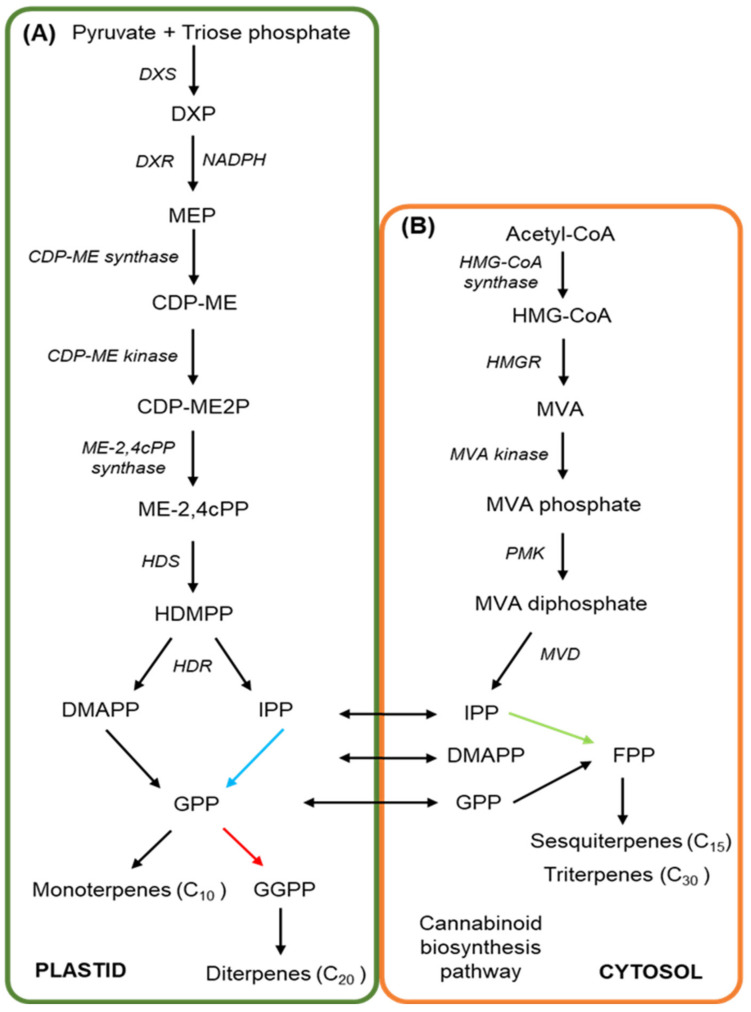
An overview of the mevalonate and methylerythritol 4-phosphate pathways in *Cannabis sativa*. The MEP (**A**) and MVA (**B**) pathways both produce terpenoid precursors, as well as the substrate for cannabinoid production, GPP. (**A**) The MEP pathway begins in the plastid with the condensation of pyruvate and glyceraldehyde 3-phosphate by DXS to produce DXP, prior to a series of enzymatic reactions to produce HDMPP. HDR then converts HDMPP to IPP and DMAPP, serving as the precursor to GPP, GGPP, and subsequently monoterpene and diterpene production. (**B**) The cytosolic MVA pathway is initiated by the conversion of acetyl-CoA to HMG-CoA and then to MVA, catalysed by the regulated, and rate-limiting enzyme, HMGR. MVA undergoes phosphorylation and then is decarboxylated to produce IPP, which is then converted to FPP as the basis for sesquiterpene and triterpene synthesis, or for GPP production for use in the cannabinoid biosynthesis pathway.

The complete biosynthetic pathway of how the prenylated polyketides, particularly minor cannabinoids, are derived from precursor molecules still requires further elucidation, particularly in view of the recent discovery of the two novel cannabinoids, THCP and CBDP [22]. Cannabigerolic acid (CBGA), the key intermediate substrate required for the synthesis of the three primary cannabinoids—cannabichromenic acid (CBCA), THCA and CBDA—arises from molecular products of the polyketide and methylerythritol 4-phosphate (MEP) pathways. A schematic representation of the MEP pathway is provided in Figure 2A. More specifically, the MEP pathway begins in the plastid via the condensation of the substrates, pyruvate and triose phosphate, a reaction that is catalysed by 1-deoxy-D-xylulose-5-synthase (DXS), and which produces 1-deoxy-D-xylulose-5-phosphate (DXP) [260,261,262]. Via the action of 1-deoxy-D-xylulose-5-reductase (DXR) in the presence of the co-factor NADPH, DXP is next reduced to MEP [263] and subsequently, MEP is converted to CDP-ME by the action of the enzyme, 4-diphosphocytidyl-2-C-methyl-D-erythritol (CDP-ME) synthase. The kinase, DCP-ME kinase then phosphorylates CDP-ME to produce 4-diphospho-cytidyl-2-C-methyl-D-erythritol-2-phosphate (CDP-ME2P) [264,265]. CDP-ME2P is subsequently converted to 2-C-methyl-D-erythritol 2,4-cyclodiphosphate (ME-2,4cPP) via the activity of the enzyme, ME-2,4cPP synthase, prior to another synthase, 4-hydroxy-3-methylbut-2-enyl diphosphate synthase (HDS), converting ME-2,4cPP to 1-hydroxy-2-methyl-2-(E)-butenyl 4-diphosphate (HDMPP). In the final step of the MEP pathway, HDMPP is used as a substrate by 4-hydroxy-3-methylbut-2-enyl diphosphate reductase (HDR) to produce IPP and dimethylallyl diphosphate (DMAPP) [264,265,266].

The HDR enzyme is essential for the in planta production of IPP and DMAPP, with over 98% of these two molecules produced by the MEP pathway. IPP and DMAPP both form essential precursor substrates for the biosynthesis of cannabinoids and terpenoids [261]. In the cytosol, IPP is also produced by the MVA pathway (Figure 2B). At the start of the MVA pathway, acetyl-CoA is converted to 3-hydroxy-3-methylglutaryl-CoA (HMG-CoA) by the enzyme, HMG-CoA synthase. Next, HMG-CoA is converted to MVA in the highly rate-limiting step of the MVA pathway, a step that is regulated via the activity of 3-hydroxy-3-methylglutaryl-CoA reductase (HMGR) [267,268,269]. MVA is then converted to MVA phosphate by MVA kinase (MVK), and subsequently, MVA phosphate is converted to its diphosphate form via the activity of phospho-MVA kinase (PMK). MVA diphosphate is subsequently converted to IPP via its decarboxylation by mevalonate 5-diphosphate decarboxylase (MVD) [270,271,272]. Via the use of yellow fluorescent protein (YFP) fusion constructs, the activity of PMK and MVD has been observed in the peroxisome in *Catharanthus roseus* (Madagascar periwinkle) and *Arabidopsis thaliana* (*Arabidopsis*) to strongly indicate peroxisomal localisation of these two enzymes in planta, and not in the cytosol [270,271,273]. IPP isomerase catalyses the conversion between IPP and DMAPP, a conversion reaction that provides the building blocks for terpene biosynthesis [274,275,276]. Geranyl diphosphate synthase (GPPS) catalyses the production of the ten-carbon (C_10_) molecule, geranyl diphosphate (GPP), via the condensation of one molecule each of DMAPP and IPP [277,278]. Similarly, formation of the C_15_ molecule, farnesyl diphosphate (FPP), and the C_20_ molecule, geranylgeranyl-diphosphate (GGPP), is catalysed by their specific synthases, farnesyl diphosphate synthase (FPPS) and geranylgeranyl diphosphate synthase (GGPPS), respectively, which condense either 2 or 3 molecules of IPP together with a single molecule of DMAPP [279,280,281]. Together, GPP, FPP and GGPP form the precursors necessary for monoterpene or CBGA biosynthesis (GPP precursor), or the numerous sesqui-, di-, tri-, or tetra-terpene products (FPP or GGPP precursors) found in *Cannabis* [282,283].

**Figure 3:**
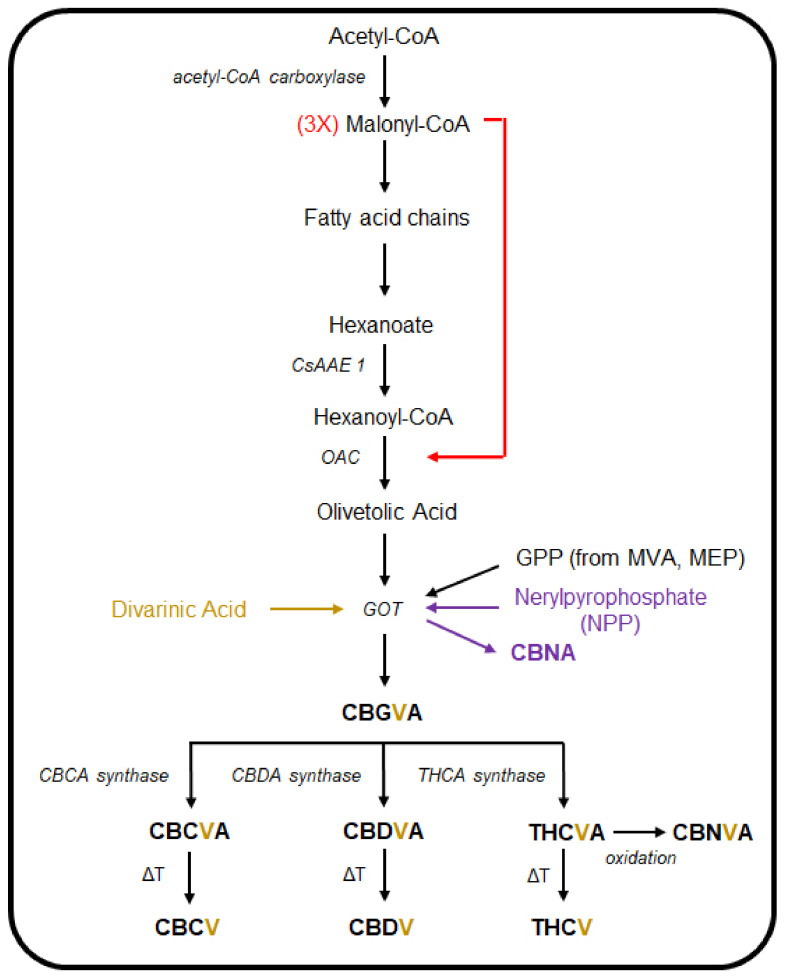
An overview of the cannabinoid biosynthesis pathway in *Cannabis sativa*. Malonyl-CoA, formed from acetyl-CoA, is used downstream with hexanoyl-CoA to produce olivetolic acid (OA). Next, OA is used as substrate along with other biomolecules by the GOT enzyme to produce the major cannabinoid precursor, CBGA. When GOT uses substrates additional to OA, such as divarinic acid or nerylpyrophosphate, a range of other minor cannabinoids are produced.

The polyketide pathway is initiated when acetyl-CoA is carboxylated to malonyl-CoA, which in turn serves as the precursor for the fatty acid chains used to produce hexanoate (Figure 3) [254,255,261]. The acyl-activating enzyme (AAE), which in *Cannabis* is encoded by two putative genes, termed *CsAAE1* and *CsAAE3*, with the encoded proteins localised to the cytoplasm and peroxisome, respectively, where they function to catalyse the synthesis of hexanoyl-CoA from hexanoate [255]. Condensation of hexanoyl-CoA, together with three malonyl-CoA molecules, is subsequently catalysed by the polyketide synthases, tetraketide synthase (TKS), or olivetol synthase [284,285]. The product of these two synthases, and post a final round of aldol cyclisation by the olivetolic acid cyclase (OAC) enzyme, is olivetolic acid (OA) [284]. Via the utilisation of GPP from the MVA pathway, OA is then prenylated by geranylpyrophosphate:olivetolate geranyltransferase (GOT), to produce CBGA [286,287,288]. The *cis* isomer of GPP, neryl diphosphate (NPP), can be used as a substrate by GOT in place of GPP, to produce cannabinolic acid (CBNA) [289]. CBGA then serves as the primary cannabinoid precursor for the synthesis of cannabichromenic acid (CBCA), THCA and CBDA, with the production of each of these three acids catalysed by a specific oxidocyclisation enzyme, namely the CBCA, THCA and CBDA synthases [289,290,291,292,293]. The use of divarinic acid as a substitute for OA by GOT, putatively produces the propyl cannabinoid homolog, cannabigerovarinic acid (CBGVA) [286,294]. The aforementioned cannabinoid-specific synthases that yield CBCA, CBDA, and THCA can all recruit CBGVA to produce cannabidivarinic acid (CBDVA), cannabichromevarinic acid (CBCVA) and Δ^9^-tetrahydrocannabivarinic acid (THCVA), respectively [294,295,296]. The resulting cannabinoids are maintained in their acidic forms until they are thermally decarboxylated to convert them into their neutral forms [297,298,299,300].

Research to date has primarily focused on the biosynthetic pathways and putative medical benefits of the two major cannabinoids, THC and CBD. Therefore, the medical and biological potential of the minor cannabinoids that also contribute to the total cannabinoid profile of the *Cannabis* plant have been largely overlooked. The small proportion that these minor cannabinoids contribute to the total cannabinoid profile of the *Cannabis* plant presents a significant obstacle for in-depth analysis of their effects when consumed. A comprehensive, and ever-increasing list of naturally occurring minor phytocannabinoids has been compiled based upon their derivation from THC, CBD, CBG (cannabigerol) and CBC, which represent the diversity that stems from variations to the three fundamental components of cannabinoids, including the (1) resorcinyl core; (2) isoprenyl residue, and; (3) resorcinyl side chain [20,301]. Eighty-two individual cannabinoids from 10 cannabinoid types, specifically the (1) CBG; (2) CBC; (3) CBD; (4) Δ^9^-THC; (5) Δ^8^-THC; (6) cannabicyclol (CBL); (7) cannabielsoin (CBE); (8) cannabinol (CBN); (9) cannabinodol (CBND), and; (10) cannabitriol (CBT) types, in addition to the miscellaneous types, and their transformation products, as well as terpenoids, hydrocarbons, sugars and fatty acids are among the constituents that comprise the chemical cornucopia of glandular trichomes. Further, several minor oxygenated cannabinoids, cannabinoid metabolites, and cannabinoid esters present in *Cannabis* have yet to be isolated and/or experimentally validated but have been identified using a variety of spectroscopic techniques [302,303,304]. In addition, a number of interesting structural formations have been observed in some of the minor cannabinoids. For example, cannabioxepane (CBX) has a tetracyclic skeleton with a seven-membered ring, a structure not previously reported for a characterised cannabinoid, while cannabisol is a Δ^9^-THC dimer with a methylene bridge. However, it must be noted that the binding affinity for specific CB receptors for these minor cannabinoids remains unknown, with some potentially not recognised, and therefore not bound by any known CB receptor [305,306]. The CBD derivative, cannabimovone, and the farnesyl prenylogue of CBG, sesquicannabigerol, were also spectroscopically characterised, with CB receptor binding assays predicting receptor–cannabinoid affinity, highlighting the structural and potential psychoactive diversity among the minor phytocannabinoids [307,308]. In addition to the identification of their parent cannabinoid precursors, plausible biochemistry behind the synthesis of these compounds is offered. However, the actual enzymatic production of many of these minor cannabinoids remains to be determined. Furthermore, the non-enzymatic formation of some of the minor cannabinoids is certainly likely, but it remains of interest to understand whether there is a greater portion of enzyme-catalysed reactions in the production of the minor cannabinoids, or indeed whether there are alternative pathways, or even additional pathway entry points in the biosynthesis of cannabinoids, both minor and primary.

## 4. Minor Cannabinoids and Their Biological Interactions

There is mounting evidence that the minor cannabinoids described above share combinations of many of the same molecular targets as THC and CBD, and therefore may potentially have unique medical applications that cannot be achieved by THC or CBD alone. The THC propyl homologue, THCV, is a CB_1_ and CB_2_ competitive antagonist against CP55,940 and WIN55,21–2, acting with similar potency to that of THC [309,310]. THCV also antagonised anandamide and methanandamide in mice vas deferens, attenuating stimulated contractile responses [309]. More recently, THCV was shown to similarly displace CP55,940 from CB_1_ and CB_2_ in CHO cells, and contrary to previous assumptions, was shown to be a weak partial CB_1_ agonist at high doses [179]. Moreover, Zagzoog et al. [310] showed THCV to produce anxiolytic, hypothermic, anti-nociceptive, hypolocomotive, and cataleptic effects in vivo in mice. CB_2_ agonism by THCV was demonstrated to reduce inflammation and attenuate hyperalgesia in mice following injection of carrageenan and formalin, respectively [180]. Neuroprotective properties were observed in 6-hydroxydopamine lesioned rats, where THCV administration preceded maintenance of tyrosine hydroxylase-positive neurons in this Parkinsonian model [311]. Similarly, THCV delayed onset of abnormal involuntary movements associated with Parkinson’s disease in mice, and reduced their severity after administration following symptom onset [181]. The in vitro demonstrated inhibition of GABA release by WIN55,21-2 at Purkinje cell synapses was reversed by THCV, which also prevented the action of WIN55,212–2 when used in pre-incubation [312,313]. In vitro studies of insulin-resistant human hepatocytes showed THCV restoration of insulin signaling mediated by CB_1_, while also improving glucose tolerance and increased sensitivity to insulin in mice obesity models [182]. Antiepileptic properties were also established in vitro, specifically when THCV reduced both the frequency and amplitude of epileptiform activity in rat piriform cortex slices [183]. The majority of published studies have focused on the CB_1_ and CB_2_ receptors, but the in vitro activity of THCV has been observed for the TRPV1 to TRPV4 group of receptors, as well as for the TRPA1 receptor [200,201,202]. THCV can enhance 5-HT_1A_ receptor activation to produce antipsychotic-like effects in rats [314], but does not affect other endocannabinoid system constituents such as PPARγ [315], FAAH [200], or MAGL [200]. One clinical trial in humans where THCV was administered once daily for five days followed by intravenous administration of THC suggested that THCV inhibited an increase in heart rate, protected against verbal recall impairment, and reduced the subjective psychoactive intensity induced by THC [316]. Further, THCV affects brain regions associated with reward and aversive stimuli, as well as areas associated with cognitive control [317,318]

Recently, a four-carbon side chain variant, Δ^9^-tetrahydrocannabutol (THCB), was isolated and which showed CB_1_ and CB_2_ binding affinities similar to those of THC, with in vivo mice studies suggesting potential analgesic and anti-inflammatory properties [176]. Similarly, the recently identified seven-carbon side chain variant, THCP, was shown to be able to bind to both CB_1_ and CB_2_ with 33 and 5 times greater affinity than THC, respectively, as well as to initiate catalepsy, hypothermia, analgesia, and reduce locomotion; all indications of potent full CB_1_ agonism [22]. THCA has been shown in rodent culture supernatants to reduce the abundance of inflammatory and oxidant markers [319,320], though no other research to our knowledge of this nature has been published. In addition, Δ^8^-THC has been shown to possess higher antiemetic effects than THC [188], and has been successfully trialed for repressing emesis in children [184]. Furthermore, in humans, Δ^8^-THC appears required to be administered at higher doses than THC to display a similar degree of psychoactive properties [321]. THCA is a 5-HT_1A_ agonist [322], a PPARγ agonist [323], and displays the same properties against TRP channels as does THC [200]. However, little pharmacological, pharmacokinetic, or recent safety data are available for any of these compounds.

Improvements in seizure frequency has been reported in an epileptic patient coinciding with increased CBDV serum levels, after which in vitro studies confirmed that CBDV, at least, possesses the ability to influence GABA receptors; a finding that indicates a potential avenue for anticonvulsant properties [324]. Further, in vitro analyses revealed CBDV to have anticonvulsant effects in four seizure models, namely the (1) maximal electroshock-, (2) audiogenic-, (3) penytylenetetrazole-(PTZ), and (4) pilocarpine-induced seizure models [325,326]. Using rat brain tissue samples, PTZ-induced seizures coincided with an increase in *Early growth response 1* (*Egr1*), *Activity-regulated cytoskeleton-associated protein* (*Arc*), *Chemokine* (*C-C motif*) *ligand 4* (*Ccl4*), *Brain-derived neurotrophic factor* (*Bdnf*), and *FBJ osteosarcoma oncogene* (*Fos*) gene expression [327]. Interestingly, the administration of CBDV was shown to reduce the expression of all of these genes [327]. Additional seizure studies identified TRPV1 as the potential receptor modulating anti-seizure effects via the use of *trpv1* knockout mice which showed a reduced response to CBDV [328]. Desensitisation of TRPV1, in addition to TRPV2, by both CBDV and CBD has been observed [199], while Ca^2+^ transients were induced in TRVP2-expressing HEK293 cells more potently by CBD than by CBDV. However, THC was a more potent inducer of Ca^2+^ transients than either CBDV or CBD [200]. Another study did alternately suggest that CBD was the more potent agonist of TRPV2 than THC, but this study did not include the assessment of CBDV [329]. Cannabinoid administration improved symptoms in mice models of Rett syndrome, including motor control and sociability [197], and through TRPA1, CBDV mediates anti-inflammatory effects in intestinal tissue of humans with ulcerative colitis [204]. Similar to CBD, CBDV inhibits FAAH and anandamide reuptake [200]. However, unlike CBD, CBDV does not show affinity for the CB_1_ or CB_2_ receptors [180]. CBDV may confer some benefit in patients with Autism Spectrum Disorder [330] and Duchenne muscular dystrophy [205]. CBDV did, however, fail to alleviate the neuropathic pain associated with human immunodeficiency virus (HIV) [331], and in another study, the administration of CBDV induced DNA damage in human cell lines at concentrations similar to those observed in *Cannabis* consumers [332], indicating carcinogenicity potential for CBDV. However, CBDV has been safely trialed in humans at a single 600 mg oral dose [330], and it remains to be determined whether CBDV will be efficacious for other illnesses in clinical trial.

CBG has shown partial agonism of CB_1_ and CB_2_, α_2_-adrenceptor agonism and 5-HT_1A_ antagonism, while exerting some minor anti-nociceptive and anxiolytic properties in vivo [179,333]. Mice models of inflammatory bowel disease (IBD) showed positive outcomes with CBG treatment including reductions in the level of reactive oxygen species in intestinal cells, as well as reduced nitric oxide concentration in macrophages through CB_2_ modulation [194]. Further in vivo animal studies provided evidence for neuroprotectivity against symptoms of Huntington’s disease in 3-nitropropionate treated mice, with improvement in motor function, reduction in proinflammatory marker upregulation and increased antioxidant defenses, with R6/2 mice showing a reduction in the expression profiles of several genes linked to the disease following CBG treatment [334]. Similarly, in vitro analysis of NSC-34 neuronal cells showed that CBG pre-treatment reduced both inflammation and the expression of pro-inflammatory cytokines, and inhibited cell death resulting from the cell culture medium of lipopolysaccharide (LPS) stimulated RAW 264.7 macrophages [335]. CBG shows a similar profile at TRP channels compared to CBD, with agonist properties at TRPV1 through to TRPV4, and at TRPA1, but antagonism at TRPM8 [200]. It is also an anandamide reuptake inhibitor [336], and an LPI inhibitor at GRP55 [97]. As for the propyl analogue of CBG, CBGV, very little information surrounding its clinical application exists, except to show that CBGV has activity at GPR55, TRPV3 and TRPV4 [198,201].

CBC use in a clinical setting, or in human trials, appears to be untested currently, and additionally, cannabichromevarin (CBCV) currently has even fewer studies dedicated to it. However, CBC has seen some use in animal models and in vitro studies. CBC has been shown to inhibit FAAH, MAGL, and anandamide reuptake [200,337], but has been demonstrated to have no effect at TRPV1 or TRPV2. Further, CBC is a very weak CB_1_ agonist [338,339,340], and only exhibits modest agonist properties at CB_2_. An early study suggested that CBC, CBCV, and a CBC variant which lacks a carbon side chain, possessed anti-inflammatory properties in rat edema models and varying anti-bacterial and anti-fungal properties [341]. More recently, CBC was seen to produce anti-inflammatory effects in LPS paw edema models in mice in CB_1_- and CB_2_-independent pathways and also produce hypothermia, catalepsy, and locomotor suppression [342]. The authors went on to suggest that the effects of CBC were altered in the presence of THC, with an additive effect against inflammation [342] and similarly, tail-flick tests revealed that subtle analgesic properties of CBC were potentiated by its combination with THC [343]. Selective CB_2_, but not CB_1_ agonism, was exhibited by CBC on mouse pituitary tumour cells, and the persistent administration of CBC caused desensitisation of CB_2_ receptors [344]. Intestinal studies suggest that CBC confers some benefit against inflammation. However, this was potentially independent of CB_1_, CB_2_, or TRPA1, the expression of which were all downregulated in the presence of CBC in one study, but shown to be unchanged in another study [193,345]. Colorectal cancer cell viability was attenuated through TRPM8 antagonism by CBG, as well as by the administration of CBD, CBDV, and CBC, albeit to lesser degrees [203]. Other studies have indicated that CBC is not a potent antagonist of TRPM8, and instead suggest that CBD, CBG, THC, and THCA are more effective antagonists of TRPM8 [200,202]. Additionally, CBC, CBN, THC, THCV, THCA, CBDA, and CBG all induced intracellular Ca^2+^ increases in HEK293 and rat DRG neurons through TRPA1 [200,202]. CBC has also shown promise in increasing neural stem cell viability in animal models (in vitro), mediated through ERK phosphorylation [346]. However, it is concerning that large amounts of CBC are required to produce pharmacological effects [90], which implies that CBC may be difficult to implement in a human health context.

The binding affinity of CBN, and of its primary derivatives, to the two main cannabinoid receptors was established in 2000, and showed rather unsurprisingly that alterations at carbon atom positions 1, 3, and 9, resulted in significantly different affinities at both receptors [347]. An earlier study indicated CBN to have cataleptic, hypothermic, and locomotive effects, as did 11-hydroxy-CBN; a hepatic microsome CYP2C- and CYP3A4-catalysed metabolite [348,349]. Additionally, CBN directly inhibited the activity of the human cytochrome P450 family 1 (CYP1) enzymes, CYP1A2 and CYP1B1 [350]. Assays of cultured neuronal cells expressing an inducible disease conferring huntingtin (Htt) protein, suggest that CBN has protective effects against cell death in vivo, with low toxicity even at the high concentrations required for protectivity [351]. Interestingly, cannabinoid receptor loss has been indicated as a pathophysiology of Huntington’s disease [352,353], which may suggest that the purported protective action of cannabinoids is independent of cannabinoid receptor binding. Subcutaneously delivered CBN delayed the onset of amyotrophic lateral sclerosis (ALS) symptoms in murine models but failed to affect survival, so was postulated to mask the early spasticity associations without affecting disease progression [354]. A synergistic effect of CBN with CBD at reducing mechanical sensitisation in rat masseter muscles was observed in one study, however high concentrations of CBD ameliorated the efficacy of CBN [355]. CBN has been reported to have no effect at FAAH, MAGL, or TRPV1, but acts as an agonist at TRPA1 and TRPV2 [200].

Via the use of in silico analyses, the even lesser-known cannabinoids, cannabiripsol (CBR) and CBT, are predicted to have cytochrome P450 inhibitor activity [356]. In another in silico study, CBL, CBT, and CBE were assessed, and ranked in this order, to have acetylcholinesterase-inhibiting function. However, their inhibitory effects were less than those of THC, CBN, and CBDV [357]. Exactly how well in silico studies translate to clinical relevance, or even to in vitro and/or in vivo studies, restricts what conclusions can be accurately drawn. Minor phytocannabinoids do represent an understudied portion of the *Cannabis* plant. Very few studies exist that have utilised an in vivo approach to ascertain the viability of minor cannabinoids to potentially produce any significant medical benefits, and fewer still cover any human clinical trials. There has been indication that some cannabinoids exhibit synergistic action, and as a result there may be value in investigating the interactions among cannabinoids or constituents of the *Cannabis* plant.

## 5. Directions in *Cannabis* Development for Secondary Metabolite Production

The establishment of superior varieties of *Cannabis* has been the target for plant breeders since the domestication of this species. To produce new medically relevant *Cannabis* varieties with elevated concentrations of specific minor cannabinoids, or to develop techniques to manipulate the cannabinoid biosynthetic pathway in other organisms, a deeper understanding of the genetics of the *Cannabis* plant is first required. Here we outline the progress in relation to (1) the sequencing of the *Cannabis* genome, and (2) the potential to molecularly manipulate the *Cannabis* plant itself for the altered production of specific cannabinoids. In this regard, we highlight the established success in *Papaver somniferum* (opium poppy), as a parallel example for maximising yield and the concentration of key secondary metabolites of medical and commercial relevance.

### 5.1. Next-Generation Sequencing of the Cannabis Plant and Its Potential for Genetic Manipulation

Over the last 25 years, various experimental approaches have been employed to unveil the wealth of information contained in the *Cannabis* genome. Using early DNA sequencing and karyotyping techniques, the X and Y sex chromosome characteristics of *Cannabis* were uncovered, as were the diploid (2n = 20) genome sizes for male and female plants [358,359]. The female *Cannabis* plant was revealed to have a genome size of 818 megabase (Mb), while the male *Cannabis* plant was determined to have a larger genome size of 843 Mb; specifically due to the larger size of the Y chromosome, compared to the X chromosome of female plants [358]. Microsatellite markers have been employed as a tool for DNA typing *Cannabis*, and these polymorphic short tandem repeat (STR) markers have been utilised as a measurement of genetic relationships among cultivars [360,361,362]. More recently, the rapid change in technologies surrounding Next-Generation Sequencing (NGS) platforms has meant that studies can unravel whole genomes in a fraction of the time required via the use of older methods. As a result, the first draft *Cannabis* reference genome, and transcriptome, were constructed in 2011 using the high THCA, low CBDA cultivar, ‘Purple Kush’, and the high CBDA, low THCA hemp strains, ‘Finola’ and ‘USO-31’ [363]. Using a PacBio long-read sequencing platform, the Purple Kush and Finola genomes were again sequenced in 2019 to generate a physical and genetic map for *Cannabis*, and further distinguish the genes, and importantly the gene products (specifically, the encoded enzymes), underpinning the secondary metabolite profiles responsible for the divergent chemotype between hemp and marijuana cultivars [364,365].

Earlier work surrounding the chemotypic variance of cannabinoids observed in *Cannabis* unveiled the relationship between THCA and CBDA synthase expression, describing a single locus (*B*), with two codominant alleles, *B_D_* and *B_T_* [295]. A 1:1:2 segregation ratio results in the production of three chemotypes of the *B* locus, including the (1) pure CBD (*B_D_*/*B_D_* homozygote), (2) pure THC (*B_T_*/*B_T_* homozygote), and (3) mixed CBD/THC (*B_D_*/*B_T_* heterozygote) chemotypes [295]. However, later studies based around NGS platforms indicated an alternate genetic model of synthase gene duplication and rearrangement at multiple linked loci, and that CBDA synthase is more ancient, has a greater affinity for the CBGA substrate, and that the CBDA synthase locus is solely responsible for the cannabinoid chemotypes observed in *Cannabis* [363,365,366,367,368,369]. In an attempt to classify variability in chemotypes, and to associate genotype to chemotype in a diverse germplasm collection, DNA sequence characterised amplified region (SCAR) markers associated with THCA/CBDA synthases were assessed in 22 *Cannabis* varieties representing 2 fibre and 1 drug type plants from East (n = 8), Central (n = 1), and South (n = 2) Asia, as well as from Europe (n = 7) and of mixed (n = 4) domestication status [370]. This approach revealed a variability in cannabinoid profiles (CBD:THC) across ‘chemotype II’, or *B_D_*/*B_T_* equivalent plants, more than three-fold greater than previously observed, supporting the allelic variant and multiple loci prediction, when assuming that a heterozygote plant in a single locus model would have a 1:1 CBD:THC ratio [370].

Other large-scale genetic diversity studies using NGS, and which compared the evolutionary relationships between 340 *Cannabis* varieties from existing datasets, and from other novel multiplexed libraries, highlighted the murky ancestry of the *Cannabis* plant resulting from generations of repeated rounds of selective breeding, and also provides an extensive data platform for future genotyping efforts [371]. Moreover, Lynch et al. [371] classed their assessed *Cannabis* varieties into three genetic groups, including (1) hemp, (2) narrow leaflet, and (3) broad leaflet drug types, in order to determine the genomic and genetic variation of their population for the potential use of varieties from each group in either agricultural or medicinal applications. The authors indicated unique cannabinoid and terpenoid profiles for each group, structured loosely around geographic origin of each species, and noted the requirement for the inclusion of the putative *Cannabis* species, *C. ruderalis*, in future studies to fully elucidate their genetic distinction and ancestral lineage [371]. The development of expressed sequence tag simple sequence repeat (EST-SSR) markers to assess genetic diversity of 115 *Cannabis* genotypes also revealed geographical-based clustering into 4 groupings, including the Northern China, Southern China, Central China and Europe groupings [372]. Interestingly, a genetic similarity coefficient derived from 45 of 117 randomly selected EST-SSRs markers revealed that despite physical proximity to the other Chinese varieties, Northern Chinese varieties had a greater similarity coefficient to the European grouping, predicted to be related to latitude and day length [372]. The analysis of inter simple sequence repeats (ISSR) of 27 native Chinese hemp varieties identified a similar geographic distribution to genetic distance relationship, while also revealing the hemp varieties were genetically diverse, yet primitive, a finding which adds further weight to the suggestion that the *Cannabis* plant originated in southern China and then spread north [373].

The recent assembly and annotation of the mitochondrial genome of *Cannabis* using NGS methods will also allow for similar studies to be performed to determine the extent of the genetic diversity among *Cannabis* varieties [374]. In addition, the assembly of two chloroplast genomes from different *Cannabis* varieties will aid in validating the phylogenetic relationship of *Cannabis* among the Rosales order of the Plantae kingdom [375]. However, as with all sequencing, repeated efforts across diverse genotypic populations compared against reference genomes will increase the accuracy and reliability of publicly available repositories. RNA sequencing as a tool for differentiating strains has been used with some success, where the transcriptome isolated from cannabinoid-containing glandular trichomes from different varieties allows for comparative analysis based on the cannabinoid and terpenoid chemical profiles [376,377]. As the regulatory landscape surrounding the use of *Cannabis* evolves, and the value of the unique chemical profile of specific *Cannabis* varieties is realised, breeders are likely to use these sequencing techniques to rapidly characterise and protect their ‘strains’. The development of such highly targeted databases provides the platform for precise manipulation of phenotypic or chemotypic traits in *Cannabis* to deliver improved medical efficacy or novel therapeutics.

A forward and/or reverse genetics approach with the application of chemical mutagenesis agents, such as ethyl methanesulfonate (EMS), a mutagen that introduces point mutations into the plant genome, is an effective approach for functional genomic assessments and effective plant breeding regimes, and has been successfully demonstrated in a variety of plant species, including hemp [9,378,379,380,381,382,383]. The application of alkylating agents such as EMS in a time-dependent manner causes a larger number of point mutations across the genome, compared to an irradiating method such as X-ray, or fast neutron bombardment, both of which produce much larger genome deletions and/or chromosome rearrangements [384,385,386]. Deletions ranging from 0.8 to 12 kilobases (kb) were produced in *Arabidopsis* using fast neutron bombardment, a widely used model plant species with an average gene density of one gene per 4.8 kb. The size of the genome alterations produced by this approach can, however, potentially cause the loss of function, or significantly altered expression of more than one gene. Therefore, a considerable drawback of using such an approach is the time and effort required post-mutagenesis to identify a ‘causative mutation’. While the *Arabidopsis* genome is comparatively smaller than that of *Cannabis*, a similar post-mutagenesis investigative strategy would likely be required in other plant species with nuclear genomes either of a similar or significantly larger-size [385,387]. Regardless, these types of methods require rather large numbers of plants to be effective as deletions and point mutations are not site directed, which is a considerable limitation as even rapid standard screening techniques demand intensive laboratory work [388,389,390].

Since the advent of the CRISPR/Cas9 gene-editing system in late 2012 [391], the ability to manipulate plant genomes has become more cost efficient and less experimentally tedious when compared to the traditional genetic engineering approaches used by plant breeders in other crop species [392]. The CRISPR/Cas9 system effectively directs site-specific genome editing using RNA-guided, microbial-derived nucleases that initiate double-stranded DNA breaks in eukaryotic and bacterial systems [391,393]. The specificity of this system greatly reduces the amount of off-target genome alterations compared to more traditional transformation techniques. However, off-targeting has also been observed with CRISPR/Cas9 use, an inherent challenge when manipulating any biological system [394,395,396,397]. Earlier work was directed towards human applications, but increasingly this system has been utilised in plant systems, with examples in *Arabidopsis*, tobacco (*Nicotiana tabacum*), rice (*Oryza sativa*), lettuce (*Lactuca sativa*), maize (*Zea mays*), soybean (*Glycine max*) and wheat (*Triticum aestivum*) now documented [398,399,400,401,402,403,404,405,406,407,408,409,410]. By no means an exhaustive list of CRISPR/Cas9-facilitated manipulation in plants, the above does, however, highlight the potential applicability of this targeted mutagenesis approach to modulate specific biosynthetic pathways in *Cannabis* to produce superior varieties that display phenotypic and chemotypic traits of interest, and as a tool to discover key genes involved the production of minor cannabinoids. Transformation technologies has thus far been conducted in hemp varieties only, and therefore require further development and considerable refinement for application in other *Cannabis* varieties. The first report of successful hemp transformation emerged in 2001 [411], and two years later, a protocol for successful *Agrobacterium tumefaciens*-mediated transformation of tissue cultured hemp callus was implemented [412]. More recently, Wahby et al. [413,414] successfully transformed hemp using both *A. tumefaciens* and *A*. *rhizogenes*, establishing the initial protocol for hairy root culture in *Cannabis*, a system used for the production of key phytochemicals. Despite these successes, *Cannabis* has proven to be a difficult plant species to transform with such variables as variety, plant age and the explant used for callus production, all demonstrated to be crucial factors underpinning transformant regeneration efficiency [415]. As with any novel plant transformation system, in order to overcome poor transformation efficiency, optimised protocols with respect to culture media, experimental approach, and selected explant material, will be required for routine and robust transformation of *Cannabis*.

### 5.2. Synthetic Production of Cannabinoids

Recently, the synthetic biology approach utilising microorganisms to produce high-quality cannabinoid products has removed the requirement for plant material [287,416]. Luo and colleagues [287] were successful in producing CBG, CBD, THC and Δ^9^-THCV from galactose, via manipulation of the native MVA pathway of the yeast *Saccharomyces cerevisiae* post the introduction of *Cannabis* genes encoding cannabinoid synthases, olivetolic acid synthase and geranylpyrophosphate: olivetolate geranyltransferase. Production of THCA from CBGA through functional THCA synthase expression in the two yeast species, *S. cerevisiae* and *Pichia pastoris*, has been demonstrated. However, attempts to introduce the same functionality in *Escherichia coli*, a bacterium, have proved unsuccessful [293,417]. Over-expression of genes encoding enzymes in the MVA and prenyl diphosphate pathways, also in *S. cerevisiae*, produced prenyl alcohol precursors required for terpenoid and cannabinoid synthesis [418], while expression of a functional aromatic prenyltransferase from *Streptomyces* resulted in THCA production from OA and DPP in the yeast, *Komagataella phaffi* [419]. These approaches present an attractive alternative with the ability to conceivably produce large quantities of minor cannabinoids that are only found in trace amounts in planta, while also reducing and/or removing the costs, carbon emissions (associated with indoor growth; [420]) and environmental variables associated with the agricultural crop production. However, it should be noted that due to the criminalisation of *Cannabis* since the early 1930s, there are very few studies analysing water and energy use associated with the cultivation of *Cannabis*, although undoubtedly, as research in this area becomes more prevalent, efficient horticultural practices will reduce the consumption of water and energy for the large-scale cultivation of *Cannabis*.

### 5.3. Phenotypic Parameters Affecting Cannabis Yield and Potency

In *Cannabis* plants exhibiting an illicit drug chemotype (high THC), a primary concern, in conjunction with desired cannabinoid content, is overall biomass yield of female floral tissue. Consistent with other agriculturally significant species, *Cannabis* is sensitive to environmental variations which alter physiological characteristics affecting plant growth and yield potential. Early work on *Cannabis* flowering, uncovered the response to photoperiodism [421,422], which has subsequently been exploited, particularly by illicit indoor growers, who can cultivate *Cannabis* year-round by manipulating the response to reduced photoperiod length [423]. Photoperiodism is a well-known biological response critical for development of branching and floral architecture in *Cannabis*, and as a result, has implications for yield potential [423,424]. A reduction in day length from 18 to 12 h induces flowering, and maintenance of this regime for 8 weeks produces an acceptable floral yield [423]. Elevated light intensity from 400 watts per square metre (W m^−2^), to 600 W m^−2^, produced a higher yield of floral tissue per plant in several chemotypes when grown indoors [424]. In addition, an increase in plant density from 16 to 20 plants m^−2^ reduced biomass yield of floral tissue in all 600 W m^−2^ treated plants [425]; a finding that indicates that light interception is compromised at the lower canopy level in crowded growth conditions. The use of different artificial lighting systems in controlled environment greenhouse applications also affects yield, but there are ‘trade offs’ when using light emitting diode (LED), versus high-intensity discharge (HID) light sources. HID lighting is generally of lower cost and generates greater photon flux density between 400 and 700 nm, while LED lighting has greater configurability for specified needs and emits substantially less heat than HID lighting; with both lighting options having similar electricity to photosynthetic photon conversion efficiencies, expressed as, µmol J^−1^ [426,427,428]. The importance of light quality has been demonstrated in cucumber (*Cucumis sativus*) where a significant increase in dry weight was measured in plants grown under an ‘artificial solar spectrum’, produced by sulfur plasma and quartz-halogen lamps irradiating a light spectrum that emulated standard sunlight, when compared with those plants provided with either fluorescent or HID lighting [429]. Photosynthetic photon flux density significantly affects harvestable floral biomass yield, while elevated UV-B radiation and electrical lighting power density (W m^−2^) increased the ‘potency’ of *Cannabis* through an elevation in THC concentration; all of which highlight the importance of light quantity and quality capture by the photosynthetic apparatus of this species to improve the harvestable output of cultivated *Cannabis* [423,430,431,432,433].

Manipulating temperature conditions in indoor growth facilities has revealed a relationship with factors affecting plant growth and development. Rate of photosynthesis, water use efficiency, rate of transpiration, and leaf stomatal conductance, all increased in *Cannabis* plants with a temperature increase from 20 to 30 °C, suggesting an optimal temperature range for cultivation [431]. Temperature and photosynthetic rate are tightly linked with the photosynthetic apparatus sensitive to fluctuations in temperature, responding particularly with reduced Ribulose 1,5-bisphosphate (RuBP) regeneration and lowered stomatal aperture, which together decreased CO_2_ uptake; both rate reducing outcomes [434,435,436]. It is worthwhile to note that *Cannabis* varieties are similarly sensitive to temperature where photosynthetic rate, water use efficiency, leaf number, and stem elongation, are modulated in response to temperature change [431,437,438]. Mineral supplementation via fertilizer application has produced mixed results in terms of biomass and secondary metabolite concentration and/or profile composition in *Cannabis*. *Cannabis* was shown to be sensitive to nitrogen (N), phosphorus (P) and potassium (K) (NPK) supplementation, as well as the plant biostimulant, humic acid. The application of NPK reduced THC, CBN and CBD content, but increased CBG content in the *Cannabis* inflorescence, while the application of humic acid was found to significantly lower the THC, CBD, CBG, THCV, CBC, CBL and CBT content of the *Cannabis* inflorescence [439]. However, N supplementation alone increased hemp seed yield, plant height, chlorophyll content, while decreasing fibre yield [440]. The application of exogenous hormones during distinct developmental phases of *Cannabis* growth has also produced mixed results in relation to secondary metabolite content and biomass. Gibberellic acid (GA) application to whole flowering plants with developed, resinous trichomes reduced chlorophyll levels, DXS activity, mono- and sesquiterpene levels, and THC content, while increasing HMGR activity, to suggest a degree of interference (either directly or indirectly) by GA to both the MVA and MEP pathways [441,442]. Abscisic acid (ABA) application at the vegetative stage of *Cannabis* development, increased chlorophyll *a* content, but reduced HMGR, THC and CBD content. In contrast, ABA application at the flowering stage of development decreased total chlorophyll and HMGR content, and increased DXS activity and the content of THC in the flowers of female *Cannabis* plants, findings which again indicated either direct or indirect phytohormone-mediated interference of both the MVA and MEP pathways [441,442].

Alterations of the architecture of the *Cannabis* flower via the application of molecular-assisted breeding, or genetic engineering, are potential strategies to increase the floral yield of *Cannabis*. Alternatively, directed manipulation of the biosynthetic pathways by application of similar approaches leading to increased cannabinoid or terpenoid content would provide greater value via the targeted elevation of the exact concentration of specific secondary metabolites. Currently, research describing the implementation of such strategies in *Cannabis* are scarce. However, investigations of trichome development in *Arabidopsis* and other plant species are not. The extremely well-annotated genome of *Arabidopsis*, combined with the ease that *Arabidopsis* can be genetically manipulated, identifies *Arabidopsis* for use in baseline studies that are potentially applicable to more valuable agricultural species. Indeed, *Arabidopsis*-based studies of trichome development have revealed a cohort of genes of interest. As with the development of any specialised cell type, it is underpinned by a complex gene network, and in *Arabidopsis*, the protein products encoded by the *GLABROUS1* (*GL1*), *GL2, GL3* and *TRANSPARENT TESTA GLABROUS* loci are responsible for various aspects of trichome morphogenesis, maturation, branching and spatial variation [443,444,445,446]. Additional gene products have been identified as essential for correct branching patterns and trichome responses to hormones, with EMS-induced mutation to the MYB encoding gene, *TRIPTYCHON*, resulting in the ‘nesting’, or grouping of trichomes with higher local densities [447,448]. A gene encoding a zinc-finger transcription factor from *Arabidopsis*, *GLABROUS INFLORESCENCE STEMS*, increased glandular trichome density on the leaves, sepals, inflorescence and its branches, while also increasing the content of nicotine secretion into the glandular heads when over-expressed in tobacco plants [449]. Similarly, overexpression of a serine proteinase inhibitor, *SaPIN2a*, from American nightshade (*Solanum americanum*) in transformed tobacco, significantly increased the branching and density of glandular trichomes [450]. Regulation of the expression of the gene encoding the DXS synthase 2 (*DXS2*) enzyme, which is active in the MEP pathway in *Cannabis*, and also in tomato (*Solanum lycopersicum*) via a RNA silencing approach, resulted in an increase in trichome density on tomato leaves and reduced the accumulation of the monoterpene, β-phellandrene [451]. In cotton (*Gossypium* spp.), a mutation in the *PIGMENT GLAND FORMATION* locus, resulted in the expression of the glandless phenotype: a strategy adopted to remove toxic gossypol from cotton seeds for human consumption [452]. While the opposite phenotypic outcome of increased trichome density would be the desired result in *Cannabis* experimentation, when taken together, these findings highlight the importance of targeting specific genetic networks for molecular manipulation to initiate the expression of desired and/or designer plant phenotypes.

Increasing the biomass of agriculturally valuable species is not a novel undertaking, and anthropogenic selection has perhaps inadvertently, been conducted by humans since the dawn of agriculture. Plant height is identified as a target for manipulation in relation to overall biomass yield in maize and sorghum (*Sorghum bicolor*) [453], and in *Cannabis* grown for fibre, stem length is an important parameter for fibre yield which is affected by plant density and soil N content [454,455]. The inverse is true for *Cannabis* varieties grown for their cannabinoid content, where reduced stem lengths produce a shorter overall plant stature and correlates with a greater photoassimilate input into reproductive tissues leading to the development of floral architecture with increased accumulation of cannabinoids and terpenoids [456]. Small [3] suggests that the value of drug chemotype varieties is linked to the development of ‘semi-dwarf *Cannabis* germplasm’, characterised by compact, congested flowers on short branches. Such plants ultimately produce more cannabinoids due to greater resource partitioning into floral and trichome development and are of short enough stature that they can be grown at high indoor densities where the artificial environment is readily manipulated to produce greater amounts of secondary metabolites. The combination of key phenotypic traits associated with increased secondary metabolite accumulation, including dense compact floral arrangements, and semi-dwarf stature, and with novel chemotypic traits that confer targeted medical efficacy epitomises the new varieties (chemovars) to be pursued as part of a highly focused research strategy. Similar strategies that use marker assisted breeding and EMS to provide the molecular basis to generate plants that produce elevated levels of desired compounds have been undertaken in other medically significant plant species. Quantitative trait loci mapping of *Artemisia annua* L. (sweet wormwood), a plant species which produces the anti-malarial compound, artemisinin, provided the platform for marker assisted breeding programs to increase artemisinin yield [457], and by extension, revealed both the pathway for similar research that would later be undertaken in opium poppy and the avenues for the future development of similar strategies in *Cannabis*.

### 5.4. Papaver somniferum: Potential Parallels for Future Cannabis Research

With significant change surrounding the societal views and scientific inquiry into *Cannabis* on the horizon, it is important to look at past endeavours to envisage future directions. While *Cannabis* is a unique plant for its utility, *Papaver somniferum* (*Papaver*; opium poppy) rivals the versatility seen across *Cannabis* varieties, and given its long history of human use, it is an excellent comparison to investigate. *Papaver*, otherwise known as opium poppy, is responsible for the production of the most medically significant alkaloids, including morphine, codeine, thebaine, oripavine and noscapine. These opioids accumulate in the phloem, particularly the mesocarp capsule of *Papaver* aerial tissues in specialised cells called lactifers, which join to form a latex-containing network of anastomosing vessels [458,459,460]. The therapeutic efficacy of *Papaver*-derived opioids is better understood than the secondary metabolites of *Cannabis*, and the scope of their effects is far reaching. Morphine has been utilised for decades as one of the most widely used analgesics, effective in the post-operative clinical setting [461,462,463]. Codeine has been shown to be a less effective analgesic than morphine [464,465] but has historically been accepted as the prevailing antitussive [466]. More recent evidence suggests however, that there are more effective treatments, especially for chronic coughing disorders [467,468,469]. Additionally, noscapine, another *Papaver* alkaloid, displays antitussive properties, and is also suggested to potentially mitigate stroke mortality and induce apoptosis in a broad set of cancers [470,471,472,473]. Thebaine and oripavine are not themselves used therapeutically. However, they are precursors for a wide range of semi-synthetic opioids including, but not limited to, hydrocodone, oxycodone and hydromorphone, as well as naloxone, which is interestingly employed to treat the acute effects of opioid overdose [474,475,476,477,478,479].

Given the multitude of efficacious compounds produced by *Papaver*, and the commercial value emanating from such, the desire to generate plant varieties that produce specific chemical profiles is one that is mirrored in *Cannabis*. While the latter is currently reliant on years of predominantly illicit breeding programs to produce plants with increased psychoactive properties, the development of novel *Papaver* varieties has already been established. EMS treatment of poppy seeds preceded the identification of a variety termed *top1* (*thebaine oripavine poppy 1*) which harboured a mutation leading to premature arrest of the morphine and codeine biosynthesis pathway. The resulting *top1* plants displayed a pigmented latex, and the enhanced accumulation of thebaine and oripavine, but failed to produce either codeine or morphine [480]. Similarly, a reduction in codeine 3-O-demethylase (CODM) activity, via either a viral-induced gene silencing (VIGS) strategy, or a fast neutron bombardment mutagenesis approach, yielded *Papaver* plants with enhanced codeine accumulation, but which were unable to synthesise morphine from a codeine substrate [481,482,483]. These high codeine *Papaver* varieties that harbour CODM polymorphisms, provided a basis for a marker-assisted breeding platform, and to produce *Papaver* chemotypes accumulating novel alkaloid profiles [483]. Similar actions utilising *Cannabis* may also mediate alterations to the cannabinoid biosynthesis pathways to produce varieties with elevated minor cannabinoid content. Sequencing of a high noscapine variety of *Papaver*, termed HN1, led to the discovery of a 10 gene cluster responsible for noscapine biosynthesis that was absent in either a high morphine (HM1)- or high thebaine (HT1)-producing variety of *Papaver* [484]. Generation of an F_2_ mapping population from HN1 and HM1 parents showed tight linkage of this gene cluster, revealing high noscapine-producing progeny that were homozygous for the HN1 gene cluster, while heterozygosity, or absence of the HN1 gene cluster, was associated with plant lines that produced low or undetectable levels of noscapine, respectively [484]. The identification of the *STORR* (*[S]- to [R]-reticuline*) locus led to the development of high noscapine *Papaver* varieties with a non-functioning cytochrome P450-oxidoreductase fusion protein, inhibiting the [S]-reticuline conversion to [R]-reticuline necessary for completion of the morphinan pathway [485,486,487,488]. A VIGS approach has been successfully utilised to individually regulate the expression of six genes encoding enzymes involved in the final six conversion steps of [R]-reticuline to morphine, each of which were shown to alter the major alkaloid profile [489]. An RNA silencing approach which employed a chimeric hairpin RNA to target all members of the multigene codeinone reductase family produced a non-narcotic, [S]-reticuline-accumulating variety of *Papaver* [490]. In the exploitation of the versatility of *Papaver* beyond narcotics, varieties with high food value have been established through EMS and gamma ray mutagenesis breeding programs to produce increased seed yield (5.66 g/capsule versus the 3.39 g/capsule of control plants) with elevated levels of unsaturated seed oil and no narcotic production [491]. While this is not an exhaustive list of selectively bred, or engineered *Papaver* varieties, the long-standing and successful development of *Papaver* varieties with superior phenotypic and/or chemotypic traits of interest certainly provides a reference for guiding *Cannabis* research strategies, which at present are comparatively in their infancy. Development of varieties producing high levels of alkaloid biosynthetic pathway intermediates is a promising indicator for the potential production of *Cannabis* varieties that reliably produce high levels of minor cannabinoids or intermediates in the cannabinoid biosynthesis pathway.

## 6. Conclusions

In summary, we have reviewed the current literature of several important aspects of cannabinoid research outside of THC and CBD, which dominate discussion in the *Cannabis* research field. Emerging research has begun to reveal the pharmacology and molecular targets of the minor cannabinoids. Due to the wide spectrum of molecular effects involved with cannabinoid consumption, it is clear that there are a range of medical ailments that could be addressed through endocannabinoid augmentation using secondary metabolites of *Cannabis*. Here, we have illustrated that via the utilisation of specific minor cannabinoids, which share some, but not all targets of THC and CBD, the medical reach of cannabinoid-containing pharmaceuticals could potentially be broadened. However, there are many challenges that currently impede this possibility, even outside of the international legal environment. Firstly, there is further room for significant characterisation of minor cannabinoid pharmacology, and currently, disease-orientated preclinical and clinical trials are lacking. Critically, techniques for producing cannabinoid isolates—even CBD and THC—are still in their infancy, and this remains a clear barrier to large-scale commercialisation of pharmaceutical cannabinoids. Here, we have reviewed the currently available literature which covers the processes involved in the biosynthesis of cannabinoids, as well as the techniques involved in the production of novel *Cannabis* chemotypes, including methods of improving yield that might be adopted from historically similar cases, such as the opioid industry. Based on this historical example, and the existing literature, it is likely that a molecular genetic modification approach will be applied to *Cannabis* to generate new opportunities for the improved yield of specific minor and major cannabinoids in the near future. In conclusion, there are multiple enticing and potentially profitable opportunities for commercial and academic growth in the *Cannabis* market outside of THC and CBD, and here, we highlight some of the most important current perspectives of this growing industry.
